# Introduction to this Special Issue: “Biomarker Discovery and Precision Medicine”

**DOI:** 10.20517/2394-4722.2019.42

**Published:** 2020-01-07

**Authors:** Bingliang Fang

**Affiliations:** Department of Thoracic and Cardiovascular Surgery, The University of Texas MD Anderson Cancer Center, Houston, TX 77030, USA.

With advances in genomics, transcriptomics, proteomics, and metabolomics, blooming data have been available for exploring molecular alternations in cancers. Many of these molecular alternations have been investigated as biomarkers for cancer diagnosis, prognosis, and precision therapies. It is my privilege to introduce this Special Issue of the Journal of Cancer Metastasis and Treatment, which contains four review articles and four original articles that focus on the topic of biomarker discoveries for cancer diagnosis and precision therapy.

Solid tumors are known to shed their cellular components (proteins, nucleic acids, lipids, glycosaminoglycans, and metabolites) or malignant cells themselves into peripheral blood. Some of these molecules are already used as biomarkers for cancer screenings and follow up tests in clinics^[1,2]^. The advent of new technologies in genomics, proteomics, metabolomics, and cell biology analyses has dramatically expanded the scope of circulating tumor biomarkers from traditional tumor-associated antigens to circulating tumor cells, circulating tumor nucleic acids (cell free DNA and miRNA), exosomes, and plasma proteomics. The tests on circulating tumor cells or tumor-specific nucleic acid in blood are also referred to as liquid biopsies^[3]^. Three review articles in this Special Issue describe recent advances and challenges in liquid biopsy. Lai *et al.*^[4]^ reviewed the use of membrane lipid-binding ligands in isolating subtypes of exosomes or extracellular vesicles for improvement of discovery and detection of disease-associated biomarkers in peripheral blood. Huang *et al.*^[5]^ discussed advances in developing new devices, such as microfluidics and nanotechnology, for capturing and molecular characterization of circulating tumor cells. Bookland and Kolmakova^[6]^ reviewed current advances in searching for circulating biomarkers for pediatric brain tumors, including cell-free DNA, non-coding RNA, tumor metabolites, and proteins in body fluids, such as cerebrospinal fluid, blood, and urine. On the other hand, the review article by Farlow *et al.*^[7]^ discussed applications of biomarkers in design of clinical trials. These reviews and discussions on advances and challenges in biomarker discoveries stimulate new thinking on addressing the challenges encountered in the field of cancer biomarker discoveries and precision therapies.

The authors of four original articles reported results of their research projects on the discovery of new cancer biomarkers. Vander Borght *et al.*^[8]^ described the generation and evaluation of monoclonal antibodies specific for exon 18 neural cell adhesion molecule for detecting small cell lung cancer cells. Ossoliński *et al.*^[9]^ reported their study on mass spectrometry-based metabolomics profiling of prostate cancer. They found over two hundred differentiating metabolites in urine, serum, and interstitial fluid of prostate cancer patients. The study presented by Zaichick *et al.*^[10]^ showed that contents of several chemical elements were drastically different between thyroid malignant tumors and normal thyroid tissues. Finally, Liu *et al.*^[11]^ reported the results of using visible resonance Raman spectroscopy for rapid skin cancer diagnosis.

I hope you enjoy reading the articles in this Special Issue on biomarker discoveries. I also want to thank Dina Li for her assistance in organizing this Special Issue.
